# The role of the ankle plantar flexor muscles in trip recovery during walking: a computational modeling study

**DOI:** 10.3389/fspor.2023.1153229

**Published:** 2023-07-18

**Authors:** Tayebeh Namayeshi, Raneem Haddara, David Ackland, Peter Vee Sin Lee

**Affiliations:** ^1^ Department of Biomedical Engineering, University of Melbourne, Melbourne, VIC, Australia; ^2^ Mechanical and Materials Engineering, Western University, London, ON, Canada

**Keywords:** fall prevention, balance, recovery strategy, biomechanical model, muscle force, gait

## Abstract

**Background:**

Reactive lower limb muscle function during walking plays a role in balance, stability, and ultimately fall prevention. The objective of this study was to evaluate muscle and joint function used to regain balance after trip-based perturbations during walking.

**Research question:**

How are lower limb muscles used to recover from external tripping during walking?

**Method:**

The dominant legs of 20 healthy adult participants with similar athletic backgrounds were tripped using a split-belt instrumented treadmill. High- and medium-intensity trips were simulated by deceleration of the dominant leg at initial contact from the speed of 1.1 m/s to 0 m/s and back to 1.1 m/s in 0.4 s and 0.8 s, respectively. Lower limb kinematics, kinetics, and muscle forces following perturbations were computed to pre-perturbation values using statistical parametric mapping (SPM) paired *t*-test.

**Results:**

A greater ankle dorsiflexion angle (mean difference: 5.3°), ankle plantar flexion moment (mean difference: 0.6Nm/kg), and gastrocnemius and soleus muscle forces (mean difference: 4.27N/kg and 13.56N/kg for GAS and SOL, respectively) were observed post-perturbation step despite the magnitude of the perturbation.

**Significance:**

This study concludes that adequate timely response of ankle function during a compensatory step is required for a successful recovery after tripping during walking in young healthy adults. Weakness in plantar flexors suggests insufficient ankle moments, which ultimately can result in falls. The findings of this paper can be used as a reference for the joint moments and range of motion needed to recover trips in the design of assistive devices. In addition to that, clinicians can use the estimated values of muscle forces and the pattern of muscle activities to design targeted training in fall prevention among the elderly.

## 1. Introduction

Falls are a major health concern both in young and older adults, with the World Health Organization identifying falls as “the second leading” cause of death worldwide in 2021, and 25% of people over the age of 65 experience a fall event at least once per year (1, 2). In a survey based on community-dwelling adults between the age of 65 and 69 years, 57.5% of the falls were caused by tripping, resulting in bone fractures (3) and soft tissue injuries, which ultimately lead to reduced quality of life (4). Falls in older adults are common (5) and result in serious health risks, financial burden on society, and decreased quality of life. A prevalence study in 2016 also found high fall rates (52%) among young adults (94 subjects, 18–35 years) despite their activity level (6). The same study reported that 58% of these fall incidents happened during walking which is an indication of the inherent instability of human walking. Another cross-sectional study based on the responses of 325 young healthy participants (19.9 ± 1.1 years) confirmed previously found results. They reported 48% of falls over a period of 16 weeks, among which 44% of falls were during walking of which 67% of those occurred following a trip or slip (7). Previous studies have shown that successful fall prevention is dependent on the ability of an individual to respond to and regain balance during their first step following a perturbation (8–11), which can ultimately be improved with targeted training (12–14).

**Figure 1:**
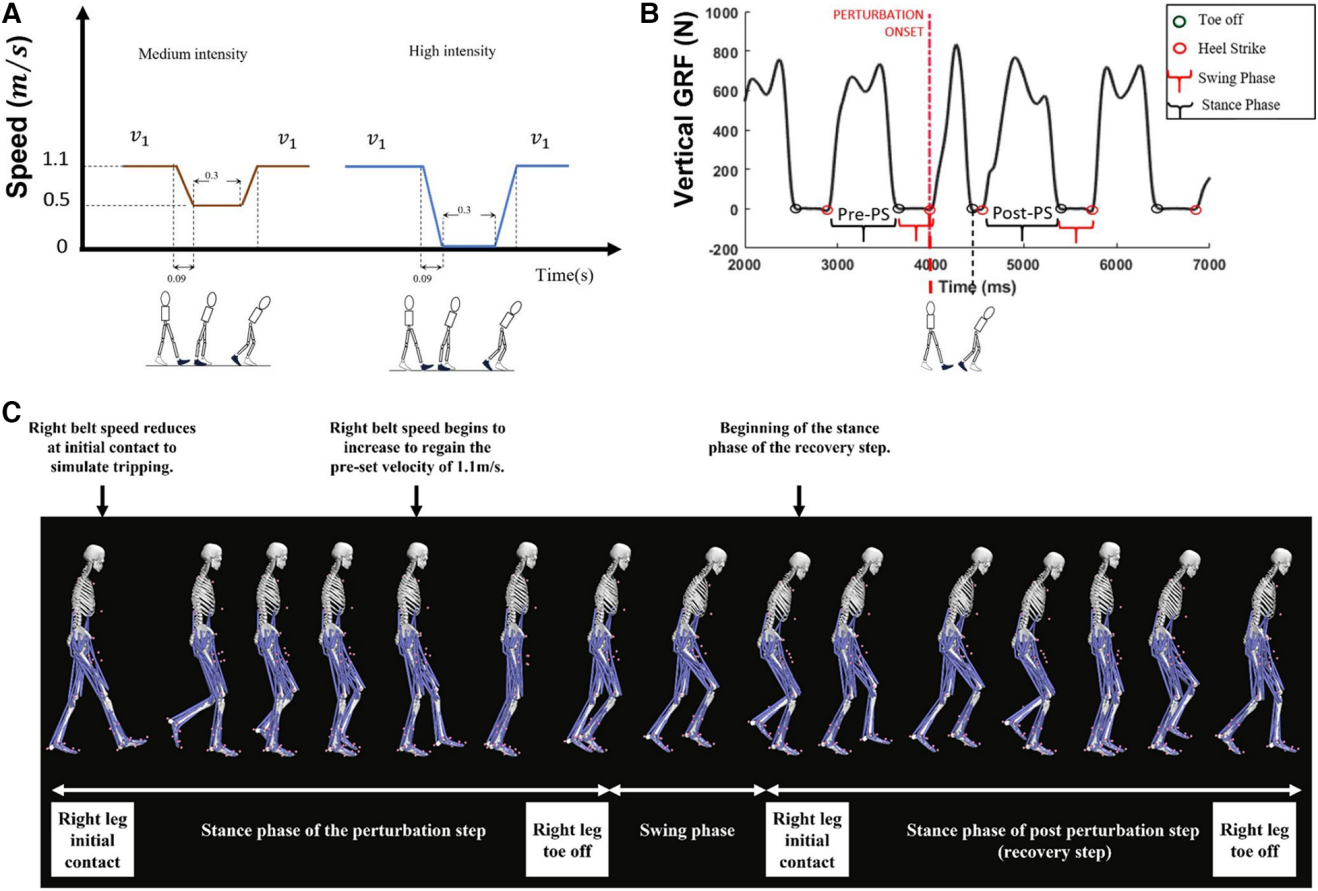
(**A**) Perturbation profile and (**B**) gait events and steps are defined in this figure. In this study, muscle and joint function between the pre-perturbation step (pre-PS) and post-perturbation step (i.e., the stance phase of the first recovery step of the dominant leg) was compared as shown in **B**. (**C**) Depiction of the anatomical skeleton model during the perturbation step and the recovery step (i.e., post-perturbation step).

Previous studies comparing the support limb during normal walking and trip recovery have reported higher peak ankle dorsiflexion and higher peak knee flexion angles in the recovery step post-tripping (11, 14, 15). Pijnappels et al. (11), during an overground obstacle–based tripping study, demonstrated that the peak knee flexion moment and peak hip extension moments were higher post-tripping, whereas Yoo et al. (1), in a treadmill-based gait perturbation study, observed no significant differences in lower limb joint moments post-tripping compared to normal walking. One study used surface electromyography (EMG) to measure changes in muscle activity following a trip and found significant increases in hamstrings (HAM), gastrocnemius (GAS), soleus (SOL), and gluteus maximus (GMAX) activity relative to that during normal walking (16). While the initial contact of the support limb following perturbation is crucial for immediate fall prevention, the subsequent step taken by the perturbed limb is particularly significant as it aids in re-establishing steady-state gait and facilitates complete recovery. In addition, the support limb's different kinematics during the recovery step compared to its normal step (11, 17) could impact the subsequent step's pattern in the perturbed limb. To date, no study to date has examined muscle force pattern differences during the recovery step of the perturbed limb (i.e., post-perturbation step of the perturbed limb, Figure 1B) following a tripping event. Our study extends the understanding of balance recovery by focusing on the post-perturbation step of the perturbed limb (instead of the support limb) as a critical component for a full recovery after a trip.

There is consensus in perturbation-based gait studies that the ankle plantar flexor muscles contribute to balance recovery after anterior–posterior perturbations while standing and walking (18, 19) and after mediolateral perturbations while walking (20). Additionally, GAS and SOL muscle activity has been reported to be lower in fallers compared to non-fallers regardless of the specific activity being performed (21); however, the dependence on ankle muscle function during the stance phase of the first recovery step post-tripping remains poorly understood. This study aimed to evaluate the role of the lower limb musculature during balance recovery from tripping by quantifying lower limb muscle forces, joint angles, and joint moments during walking both before and after a perturbation. To determine the dominance of ankle joint function during the stance phase of the first recovery step of the dominant limb following a trip, we have analyzed the joint angles and net joint moments of all lower limb joints as well as muscle forces of the main contributors to the motion of each joint. Given the known role of the ankle plantar flexors in maintaining balance through modulating whole-body center of mass position (19, 22), we hypothesized that there are significant differences in ankle muscle and joint functions during the recovery response following a trip compared to normal walking.

## 2. Materials and methods

### 2.1. Participants

A total of 10 males (age, 22.2 ± 3.5 years; mass, 71.3 ± 11.6 kg; height, 1.76 ± 0.07 m) and 10 females (age, 22.5 ± 3.4 years; mass, 57.1 ± 7.02 kg; height, 1.64 ± 0.08 m) volunteered to participate in this study. All participants had similar athletic backgrounds (performing cardio exercises for more than 3 h/week) and had no history of musculoskeletal injuries. This study was approved by the Human Research Ethics Committee (Melbourne University Ethics ID 1034932.9), and all participants gave their written informed consent prior to participating in the study.

### 2.2. Experimental protocol and data collection

Each participant walked on a dual-belt instrumented treadmill in the Computer Assisted Rehabilitation Environment (CAREN, Motekforce Link, the Netherlands). Prior to data collection, the dominancy of the leg was identified by participants' choice of using the left or right leg to kick a ball. In this study, all participants have a right dominant leg. A total of 45 reflective markers were mounted on the bony landmarks and non-landmark skin of the participants (23). Following the SENIAM guidelines (24), seven Delsys Trigno wireless electromyogram (EMG) sensors (Delsys Inc., Natick, MA, USA) were placed on the seven lower limb muscle bellies of the dominant leg in line with muscle fibers over the gastrocnemius medialis (GM), biceps femoris, rectus femoris, vastus lateralis, vastus medialis, semitendinosus, and tibialis anterior. Marker trajectory data were recorded by a 10-camera motion capture system (Vicon Nexus, Oxford Metrics) and sampled at 100 Hz. Ground reaction force data and muscle activity data were simultaneously sampled at 1,000 Hz and 2,000 Hz, respectively.

Participants were then instructed to walk at a pre-defined speed of 1.1 m/s. This speed is below the self-selected walking speed of most healthy subjects (1.35 m/s) (25), which may present a greater fall recovery challenge due to lower stability at slower speeds (26). After a 5-min treadmill familiarization period, the dominant leg was unexpectedly decelerated at initial contact with one of the two different intensities. Due to the limitations of treadmills and inherent latency of the system, the perturbation was ultimately applied at about 87 ms following initial contact (27, 28). The calculation of the delay was achieved by subtracting the time, and the triggering command was sent from the time at which the speed change was identified for multiple perturbation trials and subsequently averaging these results. Perturbation profiles were defined by a series of changes in treadmill velocity (Figure 1A). A medium-intensity trip was simulated by first decreasing the speed of the treadmill belt under the dominant leg to 0.5m/s, allowing a 0.3s of settle time, and then accelerating it to the pre-defined speed (1.1m/s) (Figure 1A, brown signal). The same procedure was used to simulate a high-intensity trip; the only difference was that the speed was decreased to 0m/s before the settling time (Figure 1A, blue signal). A minimum of 25 s washout period between each perturbation was employed. No instruction regarding the compensatory method was given to participants. Perturbations were applied randomly and without notice to the dominant limb during stance, simulating a trip during the swing of the contralateral limb. Marker data and force plate data of the normal walking and the first stepping response (Figure 1B) of individual participants were recorded for three trials of medium deceleration and three trials of high deceleration; however, to mitigate the influence of learning and adaptation, only the first perturbation was used for subsequent gait analysis (*n* = 1, refer to Appendix 1). Force plate data and marker trajectory data were initially low-pass filtered using a second-order Butterworth filter with a cutoff frequency of 6 Hz. Ground reaction force filtered data were downsampled from 1,000 Hz to 100 Hz to match the sampling rate of markers data, and the gait events were quantified (see Appendix 2). Linear envelop detection was performed on raw EMG data using a fourth-order zero-lag high-pass filter (cutoff frequency of 20 Hz), followed by a full-wave rectification and a low-pass filter (cutoff frequency of 15 Hz). The filtered data were then normalized to the maximum amplitude reached during the stance phase for each resulting linear envelope.

### 2.3. Musculoskeletal modeling

A 23 degree-of-freedom (DoF) subject-specific musculoskeletal model with 13 rigid body segments and 92 muscles was employed to perform all model analyses. The head and trunk were modeled as one rigid body segment that was attached to the pelvis *via* a 3-DoF ball-and-socket joint. Knees and ankles were modeled as individual 1-DoF hinge joints rotating in the sagittal plane. The subtalar ankle joint was modeled as a 1-DoF hinge joint to represent the inversion and eversion of the ankle in the frontal plane. Subject-specific models were scaled to the anthropometry of each individual. Joint angles were calculated using inverse kinematics (IK), and net joint moments were evaluated using inverse dynamics (ID) (29). Joint moments were then decomposed into muscle forces using static optimization, subject to constraints on muscle force–length and force–velocity relations, as well as minimizing the activation level of muscle at each time step (30).

### 2.4. Statistical analysis

The stance phase of the pre- and post-perturbation gait cycles of the perturbed leg of all trials was detected for further analysis. In this paper, the “pre-perturbation step” refers to the stance phase of the dominant leg prior to the administration of the perturbation, while the “post-perturbation step” refers to the stance phase of the first gait cycle of the perturbed limb after the perturbation is applied. The stance phase was defined from the initial contact of the right leg and to the toe-off of the same leg. All gait data were time-normalized to the gait cycle length, and muscle forces were normalized by the body mass of the subject. One-dimensional statistical parametric mapping (1DSPM) paired *t*-tests (31) were used to detect a statistically significant difference in each dependent variable (i.e., joint angles, net joint moments, and muscle forces) between the pre-perturbation step as the control group and the post-perturbation step as the study group for the medium-intensity perturbation. To test the effect of higher-intensity tripping on the recovery performance of participants, a second 1DSPM paired *t*-test was performed between the stance phases of the post-perturbation responses to the medium-intensity trip and the post-perturbation responses to the high-intensity trip. The significance thresholds were set to α<0.05. All statistical analyses were conducted using the SPM open-source MATLAB toolbox.

## 3. Results

### 3.1. Kinematics

**Figure 2:**
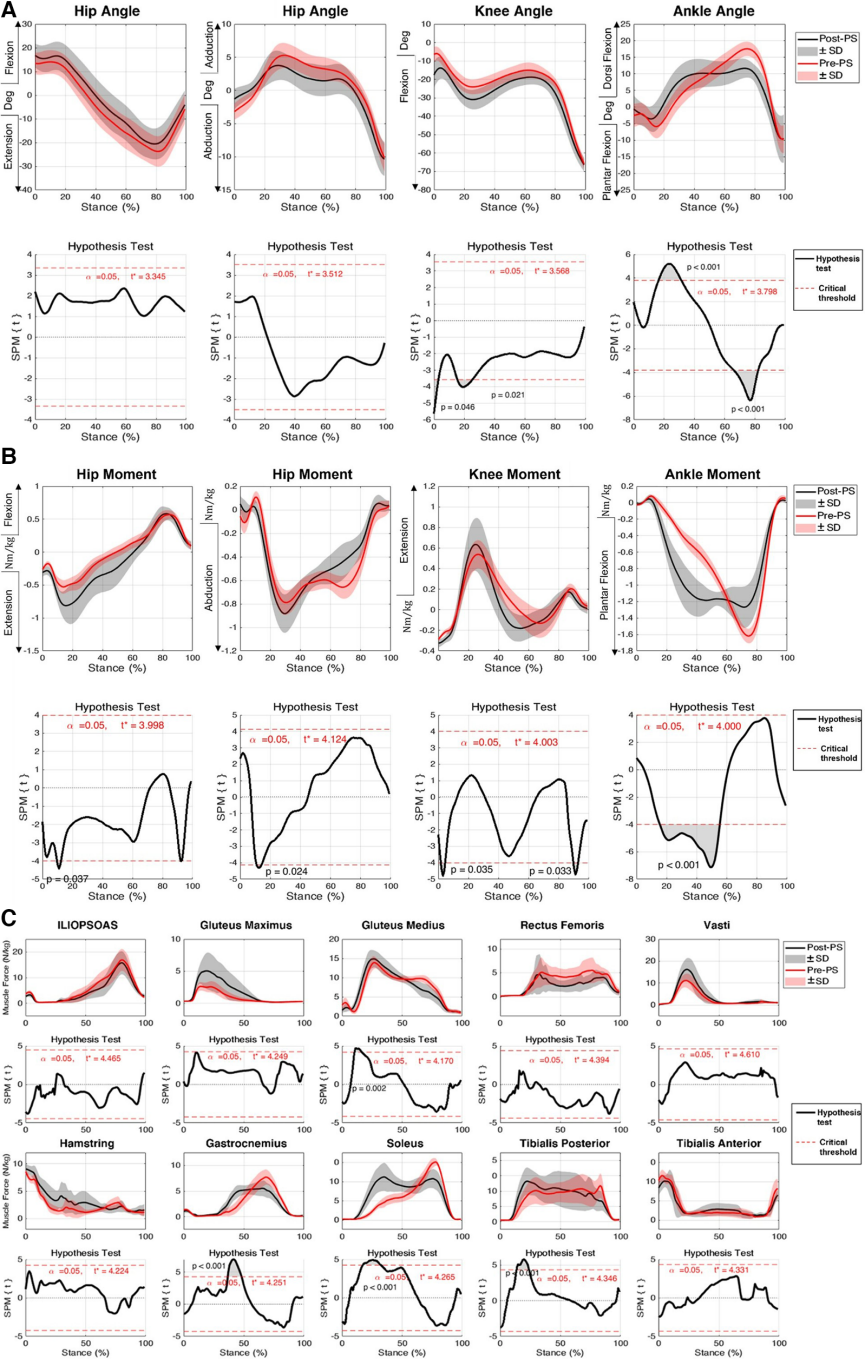
Responses of subjects to medium-intensity perturbation during the post-perturbation step (post-PS) compared to the pre-perturbation step (pre-PS) including (**A**) joint kinematics, (**B**) net joint moment, and (**C**) muscle forces. Black lines indicate the average of parameters post-perturbation, while red lines indicate the average of the parameters during the pre-perpetration step. 1DSPM{t} paired *t*-test was used to test the similarity between the magnitude and pattern of the joint angles, joint moments, and muscle forces during pre- and post-perturbation steps. Shaded areas show one standard deviation from the mean. The significance threshold was set at α=0.5.

There was no change in hip flexion angles post-perturbation throughout the stance phase. At initial contact of the foot during the post-perturbation step, an increase in knee flexion angle (mean difference: 11.1°, *p* = 0.046) was observed compared to that in the pre-perturbation step, as well as in the early stance phase between 16% and 26% (mean difference: 5.7°, *p* = 0.021). Following perturbation, during double support, the ankle was more dorsiflexed (mean difference: 5.3°, *p* < 0.001), whereas before the administration of the perturbation, at the corresponding stage of the stance phase, the ankle joint was plantarflexed. During the late stance phase, there were no statistically significant differences between the post-perturbation step and the pre-perturbation step of lower limb joint angles (*p* > 0.05), except for the ankle, which was significantly more dorsiflexed (mean difference: 6.5°, *p* < 0.001) (Figure 2A).

### 3.2. Kinetics and muscle forces

No significant differences were observed in the predicted GMAX, HAM, and VAS muscle forces when compared between pre- and post-perturbation. During midstance, a number of muscles generated significantly larger forces in the post-perturbation step when compared to those in the pre-perturbation step, including GAS (mean difference: 4.27N/kg, *p* < 0.001), SOL (mean difference: 13.56N/kg, *p* < 0.001), TIB_P (mean difference: 1.75N/kg, *p* < 0.001), and GMED (mean difference: 2.4N/kg, *p* = 0.002) (Figure 2C, Table 1).

**Table 1:** Statistical analysis results of the kinematic, kinetic, and muscle force outcome measures for medium perturbation between the pre-perturbation step (pre_PS) and post-perturbation step (post_PS) where the largest differences occurred.

	Variable	Range with statistical differences	Mean ± SD at the point of maximum difference between the pre_PS and post_PS	*P* value
Kinematics	Ankle dorsiflexion angle	17%–32%	0.2 ± 3.4° vs. 5.1 ± 4.4°	<0.001
		66%–82%	15.2 ± 2.2° vs. 8.5 ± 3.5°	<0.001
	Knee flexion angle	0%–3%	3.9 ± 3.9° vs. 17.6 ± 6.9°	0.046
		16%–26%	20.16 ± 4.1° vs. 25.9 ± 4.8°	0.021
Kinetics	Ankle plantarflexion moment	15%–56%	0.54 ± 0.10Nm/kg vs. 1.14 ± 0.24Nm/kg	<0.001
	Knee extension moment	2%–4%	0.22 ± 0.03Nm/kg vs. 0.3 ± 0.04Nm/kg	0.035
		90%–93%	0.15 ± 0.04Nm/kg vs. 0.8 ± 0.04Nm/kg	0.033
	Hip extension moment	10%–13%	0.52 ± 0.1Nm/kg vs. 0.78 ± 0.21Nm/kg	0.037
	Hip abduction moment	11%–15%	0.08 ± 0.09Nm/kg vs. 0.25 ± 0.12Nm/kg	0.024
Muscle Forces	Gastrocnemius (GAS)	37%–49%	2.01 ± 1.83N/kg vs. 6.28 ± 2.38N/kg	<0.001
	Soleus (SOL)	17%–33%	8.21 ± 2.33N/kg vs. 21.77 ± 7.12N/kg	<0.001
	Tibialis posterior (TIB_P)	14%–24%	3.5 ± 1.58N/kg vs. 5.25 ± 1.58N/kg	<0.001
	Gluteus medius (GMED)	11%–17%	1.71 ± 1.01N/kg vs. 4.11 ± 1.77N/kg	0.002

Participants exhibited significant changes in the net plantar flexion moment of the ankle joint for most of the first half of the stance phase following perturbation (mean difference: 0.6Nm/kg, *p* < 0.001). Additionally, for a brief time in the early portion of the stance phase of the post-perturbation step, a significant increase in net hip extension moment (mean difference: 0.26Nm/kg, *p* = 0.03), net hip abduction moment (mean difference: 0.16Nm/kg, *p* = 0.024), and knee extension moment (mean difference: 0.08Nm/kg, *p* = 0.035) was exhibited. The net knee extension moment was slightly decreased during the post-perturbation step compared to that during the pre-perturbation step at the end of the stance phase (mean difference: 0.07Nm/kg, *p* = 0.033) (Figure 2B). Joint motion patterns in the transverse plane showed no differences pre- and post-perturbation.

### 3.3. Effect of perturbation intensity

No statistically significant differences in the responses of the lower limb kinematics, kinetics, and muscle forces were observed as a result of change in trip intensity, except for a small increase in the magnitude of hip flexion angle (between 2% and 6%, mean difference: 4°, *p* = 0.044), hip abduction moment (between 8% and 10%, mean difference: 0.09Nm/kg), and GMED muscle force at the beginning of the stance phase (between 7% and 9%, mean difference: 1.32Nm/kg, *p* = 0.036) (Table 2 and Supplementary Figure S1).

**Table 2:** Statistical analysis results of the kinematic, kinetic, and muscle force outcome measures between post-perturbation steps of a medium and high-intensity trip.

	Variable	Range with statistical differences	Mean ± SD at the point of maximum difference between medium- and high-intensity post-perturbation step	*P* value
Kinematics	Hip flexion angle	2%–6%	15.46 ± 9.22° vs. 19.77 ± 8.42°	0.044
Kinetics	Hip abduction moment	8%–10%	0.03 ± 0.06Nm/kg vs. 0.26 ± 0.05Nm/kg	0.036
Muscle Forces	Gluteus medius (GMED)	7%–9%	1.78 ± 0.75N/kg vs. 3.1 ± 0.705N/kg	0.036

### 3.4. Validation against EMG data

**Figure 3:**
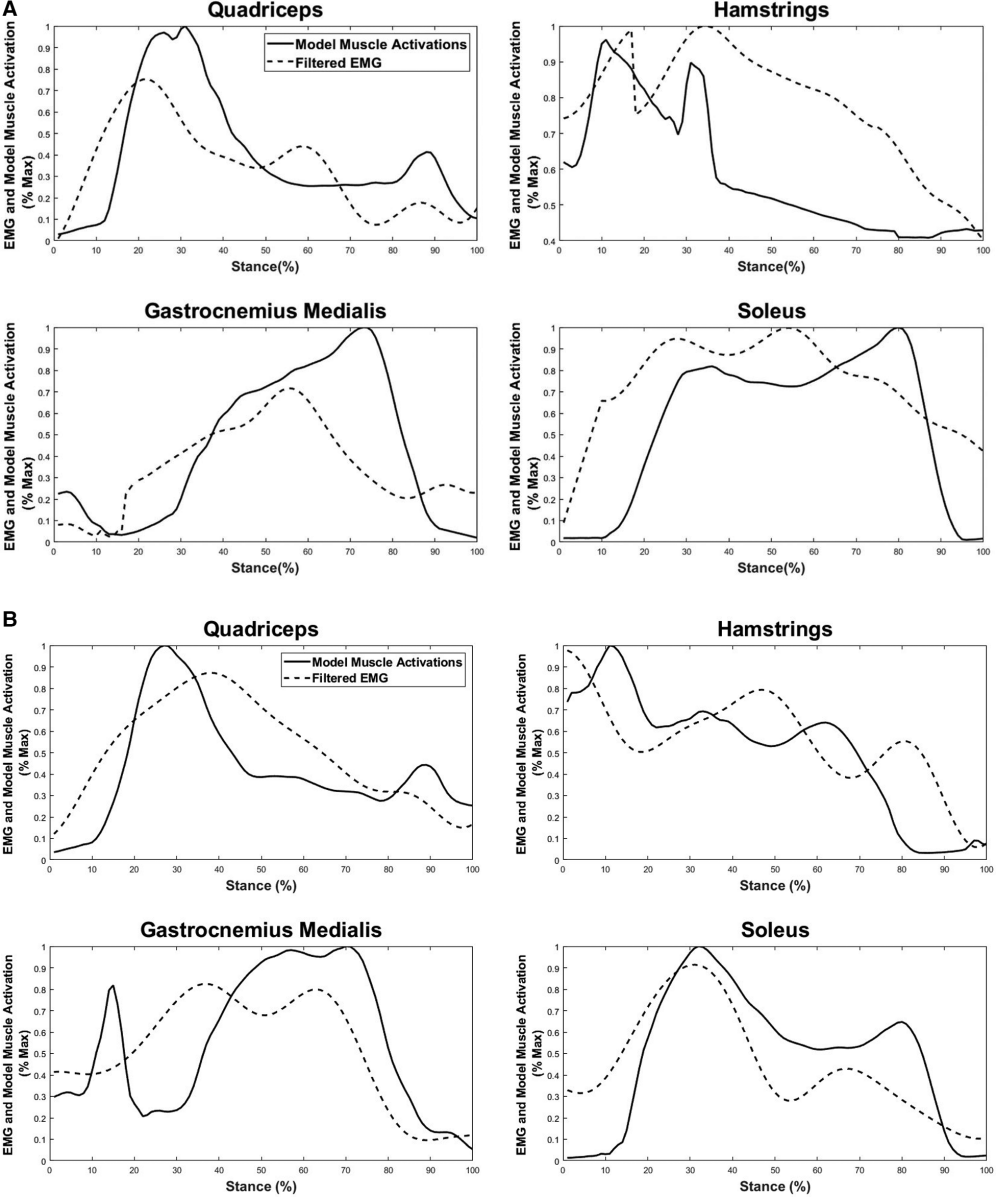
Comparison between filtered experimental EMG recordings and estimated muscle forces during the post-perturbation step in response to the (**A**) medium-intensity and (**B**) high-intensity trips. Estimated lower limb muscle activations include quadriceps (rectus femoris, vastus medialis, and vastus lateralis), hamstrings (biceps femoris and semitendinosus), gastrocnemius medialis, and soleus.

The resulting linear envelope obtained from EMG recordings showed that the onset/offset timing of the experimentally recorded muscle activities post-perturbation closely matched the estimated muscle activations during the post-perturbation step for both medium- and high-intensity perturbations (Figure 3). For medium- and high-intensity perturbation, quadriceps’ EMG first exhibited increasing and then decreasing activation during the post-perturbation step, which followed the pattern of the estimated muscle activation by the musculoskeletal modeling. HAM’s EMG demonstrated an overall decreasing activation throughout the stance phase, which is also a close match to the numerically estimated muscle activity. This indicates that the timing of muscle activation onset (non-zero values) and offset (zero values) in the experimental EMG data (non-zero values) and the estimated muscle activations have minimal differences.

The EMG data of plantar flexors [gastrocnemius medialis (GM) and SOL] also exhibited similarities for over 80% of the stance phase; however, the predicted muscle activation for GM underestimated the actual muscle activation from about 20% to 40% of the stance phase for both medium-intensity and high-intensity scenarios and an overestimation from 60% to 85% of the stance phase for the medium-intensity trip.

## 4. Discussion

The primary goal of this study was to investigate the role of the ankle joint during the stance phase of the post-perturbation step following a trip event (Figure 1B) to better understand the recovery of healthy adults from loss of balance, unlike previous perturbation studies which have focused on the step during the perturbation (32–34). Our findings confirm our hypothesis of the significant role of the ankle joint during the stepping response in regaining balance during walking after a trip. This analysis is clinically relevant, since the reduced function of the ankle muscles with age may present a risk of capacity to recover from a gait perturbation (11, 35).

Previous trip recovery studies have been mostly limited to lower limb joint function of the swinging limb (36, 37) or joint function during the stance phase of the perturbation step in a treadmill-evoked trip study (34), while lower limb joints during the stance phase of the first stepping response following the perturbation step have been shown to play an important role in the maintenance of balance (38, 39). Another study by Crenshaw et al. (40) also analyzed the first stepping response post-perturbation, albeit with a different perturbation profile consisting of a series of accelerations and decelerations, focusing on the responses to the first and last disturbances. In contrast, our study employs deceleration followed by acceleration of the treadmill to simulate tripping, emphasizing the recovery strategy taken in response to an unexpected perturbation. A previous study by Lee et al. (39) analyzed the recovery step post-tripping on a dual-belt treadmill randomly applied to the non-dominant foot but focused on maximum values in kinematics and average RMS values of four muscles obtained from EMG sensors to perform a comparison between recovery steps post-slipping and post-tripping. Our study, on the other hand, concentrates on recovery response by studying all lower limb joint angles, moments, and individual muscle forces over the entire stance phase post-tripping applied to the dominant foot.

Another reason for the significance of the stance phase of the first stepping response of the dominant leg as opposed to the perturbation step is the fact that in the treadmill-evoked perturbation studies, the perturbed leg involuntarily follows the dictated treadmill belt speed to simulate a trip as shown in Sloot's work (34). Additionally, this study is centered around the recovery process leading back to a steady-state gait, differing from studies like Pijnappels et al.’s (11) that examine immediate fall prevention responses by the contralateral leg. Therefore, the response following the perturbation includes more valuable information in understanding the full capacity of the muscles and joints to recover from loss of balance.

We showed in the first half of the stance phase during the recovery step, knee flexion and ankle dorsiflexion angles were higher at initial contact and during the single support of the dominant leg. In the second half of the stance phase, knee flexion continued to increase whereas ankle dorsiflexion angle decreased leading to less variability in the overall range of motion of this joint post-perturbation step compared to the pre-perturbation step. Greater ankle dorsiflexion and knee flexion during the early stance of the stepping response to a trip compared to normal walking may be an attempt to lower the position of the whole-body center of mass (CoM) to bring it closer to the base of support to increase stability in the horizontal direction in the transverse plane. This is in agreement with a previous study that showed the importance of lowering CoM to regain balance following belt acceleration as a recovery strategy used in healthy adults (41). Furthermore, the reduction of the ankle joint range of motion in the second half of the stance phase post-perturbation compared to the pre-perturbation step may indicate more active control of the joint stability by altering the pattern and rate of change of this joint following the trip.

Differences in the simulation of trips, analyzed steps, and post-analysis methods make a direct comparison with existing literature difficult. In a belt acceleration study, the ankle joint was shown to compensate for the loss of balance using the work–energy approach (42). We also found a significant increase in the magnitude and rate of change of ankle plantarflexion net joint moment during the stepping response, which was observed over 41% of the stance phase of gait. For the hip and knee joints, the increase in the magnitude and rate of change of joint moments occurred over a relatively short period of time (in the load acceptance phase), and the differences were less pronounced compared to those at the ankle joint moment.

During the simulated trip, the leading foot decelerates and moves backward with respect to the trunk, with the ankle joint experiencing a forward angular momentum due to the weight of the head and trunk section. To adapt to this disturbance and avoid a fall, timely corrective responses must be taken to attenuate the forward-rotating angular movement of the whole body. A previous study has shown that an increase in ankle plantar flexion moment reduces the angular momentum of the whole body (11). This may explain our finding of an increased ankle plantar flexion moment during the stepping response to a trip compared to that during normal walking. This increase could be interpreted as the attempt of the body to reduce whole-body angular momentum.

In this study, we assumed the post-perturbation step as a static optimization problem because it is computationally efficient, yet it has demonstrated validity in various dynamics tasks such as level walking (43–45), incline walking (46), running (23), drop-landing (47, 48), and post-slipping (28). In addition, the validation of computed muscle forces against EMG measurements confirmed this assumption.

Our estimated muscle activities showed a significant increase in muscle forces generated by GAS and SOL (as primary plantar flexors) and TIB_P (as the secondary plantar flexor) post-perturbation during the first half of the stance phase. Together with the previous findings of increases in the magnitude of ankle dorsiflexion angle, ankle dorsiflexion moment and ankle plantar flexors compared to pre-perturbation walking indicate that a more ankle-dominant strategy was selected by young healthy adults to recover from the simulated trip. The term “ankle strategy” in a perturbation recovery is previously defined as the response of the body to a perturbation in such a way that the ankle generates the primary forces to regain balance with little to no motion in the hip and knee joints (49, 50). Utilizing a more ankle-dominant strategy as the initial mechanism to cope with the loss of balance due to sudden lowering of the platform (51), manual removal of the platform from under the foot (52), and unanticipated bump (53) has been proven in various studies. Another research used the work–energy approach and found that in response to a sudden treadmill acceleration (slip-like perturbation), the ankle joint best reflected the changes in overall leg work (42). The similarity of our findings regarding the importance of the ankle joint during the recovery step following a trip when compared to the results of the abovementioned studies supports the finding that the human body reacts to different perturbations in a similar way by utilizing an ankle-dominant strategy as its primary choice in balance recovery. Although the concept of ankle strategy has been less explored in gait perturbations, our study findings suggest that a similar mechanism may be involved in balance recovery during gait, with the ankle joint playing a significant role in generating forces to counteract the destabilizing effect of the perturbation. Further research is needed to better understand the applicability of the ankle strategy concept during gait perturbations and its potential implications for balance recovery strategies.

There are a number of limitations associated with this study. We studied just one form of trip that was simulated on a treadmill. However, trips in daily life commonly occur when the swinging leg fails to successfully pass a physical obstacle, resulting in impact. In our experiments, trips were induced by the deceleration of the treadmill at initial contact. While other forms of trips may produce different motor responses in recovery, this method of simulating a trip has been proven to be an effective way to evoke an overground trip-like response using a treadmill (54). We also used a treadmill in a laboratory setting to achieve repeatability of testing; however, some differences in perturbation response may result if our testing protocol was employed during overground gait. Finally, although we looked at responses to different intensities of trips, the effect of speed on recovery responses was not analyzed and ought to be considered in future research.

In conclusion, successful recovery after tripping in young healthy adults during walking is associated with a more ankle-dominant strategy marked by greater ankle dorsiflexion angles, a larger peak ankle plantar flexion moment, and higher ankle plantar flexor muscle forces during the post-perturbation step when compared to those during the pre-perturbation step and other joint strategies, such as those involving the hip and knee. This finding was consistent regardless of the magnitude of the perturbation. These results provide insight into the underlying requirements of a successful recovery strategy following perturbations to walking in terms of setting reference values for required joint range of motion, joint moments, and muscle forces and may ultimately assist in the design of fall prevention interventions and assistive devices.

## Data Availability

The raw data supporting the conclusions of this article will be made available by the authors, without undue reservation.

## Appendix 1: Repeated trial exclusion criteria

After examination of the trial repetition effect across repeated trials using 1DSPM rmANOVA on joint angles and muscle forces of the right leg during the stance phase of the first stepping response of the dominant leg, results indicated evidence of differences between trials in the first stepping response on the following parameters: hip angle, ankle angle, and HAM's force (Supplementary Figure S2). This can be interpreted as learning effects after repeated trials. The purpose is to investigate individuals’ responses to an unanticipated perturbation and their responses to sudden loss of balance. Since the statistical analysis results suggest some learning over the three trials (hip angle from 0% to 17% and 33% to 65% with a critical *F* value of 5.208, *p* = 0.046 and *p* = 0.022, respectively; ankle angle from 95% to 98% with a critical *F* value of 6.467, *p* = 0.047; and HAM force from 40% to 45% with a critical *F* value of 7.963, *p* = 0.028), including them in the analysis would defeat the objective of this paper. As a result, only the recordings of the first trial for each participant were taken into account for the rest of the analysis and the repeated trials were ignored.

## Appendix 2: Hybrid gait event detection algorithm

Different strategies were observed by participants in order to overcome the loss of balance. There were two responses to the trip that led to incorrect gait event detection using traditional methods. The first scenario was when both feet landed on the same treadmill right after the administration of the perturbation. This strategy is referred to as “cross-stepping” (Supplementary Figure S3). Cross-stepping on a dual-belt treadmill led to incorrect identification of gait events by force plates. Another problematic scenario was due to latency and slow response time of force plate data, and there were situations in that participants had lifted one of their legs for a very short period of time and landed on the force plate immediately. Since GRF data was still within the threshold, using the traditional method (i.e., gait event detection solely based on GRF data) could not successfully detect a separate step.

On the other hand, marker data can be noisy, and/or missing marker data on some frames is a commonly seen phenomenon. Therefore, relying solely on markers data reduces the accuracy of event detection. Considering the significance of gait cycle detection for this study, a hybrid method was introduced.

In this method, the stance phase was determined using GRF and the velocity of the heel markers. Applying the proposed hybrid method, all gait events could be successfully detected and utilized in statistical analysis (for a simplified schematic of the algorithm, please refer to Supplementary Figure S4).

Gait events from force plate data are detected using the concept of zero-crossing with a minor modification that, here, a threshold of 10N was used instead of zero in order to filter the noise of force plate data. In addition, 10N is chosen because of a previous study that identified this value as the best cutoff threshold value for force plates (55). In this method, once the vertical component of the GRF signal exceeds 10N, it is identified as a potential initial contact event, and the moment this value is below 10N, a potential toe-off event is recorded. In the next step, the velocity of the treadmill for the same leg at those particular moments is computed using heel marker data. In this experiment, a known preset speed of 1.1 m/s was chosen for the treadmills. Later, the computed velocity was compared to the pre-defined velocity (1.1 m/s); if these two values matched, the moments obtained from the original GRF data were selected as the correct TO and HS. However, in the case of a mismatch between these two velocities, the marker data were used to identify the time stamp of the HS and TO.

In the case of cross-stepping of one of the legs, GRF data of the treadmill under that leg were below the threshold level of 10N, even though an HS had happened. In this case, a second checkpoint was implemented in the code to examine all the data points below 10N to see the possibility of cross-stepping. To examine the occurrence of cross-stepping, the sign change of the z-component of the heel marker position was used. The origin of the inertia frame was located on the line that splits the belts of the treadmill, and the blue line indicates the positive direction of the y-axis (Supplementary Figure S3). Therefore, changes in the sign of the z-component of the heel marker position are the condition to detect cross-stepping. If the gait cycle was identified as a cross-stepping, marker data were directly used to identify the HS and TO. If the cross-stepping condition was not satisfied, then GRF is below the threshold that represents a normal toe-off event.

In the event of the second problematic scenario which was due to latency and slow response time of the force plate to identify very short steps, we observed a speed mismatch between the treadmill speed and the heel marker speed, while the event was not identified as a cross-stepping in the previous checkpoint and vertical GRF was above 10N. In this case, we could conclude there was a quick lifting of the leg, and it was so fast that the force plate was unable to detect it as a separate event. The rational conclusion was the occurrence of a new gait cycle. For simplicity, this fine-tuning process is not shown in Supplementary Figure S4.
