# Supplementary material for: The role of the ankle plantar flexor muscles in trip recovery during walking: a computational modeling study
Source: Front Sports Act Living. 2023 Jul 18;5:1153229. doi: 10.3389/fspor.2023.1153229 (PMC10390771; doi:10.3389/fspor.2023.1153229)
Supplement: Supplementary file 1 [file Presentation1.zip › SupplementaryFiguresCaption.docx]

# **Supplementary figures caption**

**Supplementary Figure 1.** Responses of subjects to medium intensity perturbation compared to high intensity perturbation during the post-perturbation steps including a) joint kinematics, b) net joint moment and c) muscle forces. Black lines indicate the average of parameters following a high intensity trip, while red lines indicate the average of the parameters following a medium intensity trip. 1D-SPM{t} paired t-test was used to test the similarity between magnitude and pattern of the joint angled, joint moments and muscle forces following high and medium perturbation during post-perturbation step. Shaded areas show one standard deviation about the mean. Significance threshold was set at $\alpha=0.5$

**Supplementary Figure 2.** 1D-SPM{F} 1way repeated measure ANOVA across trials on right leg during stance phase of the first stepping response after tripping indicates learning effect on three parameters: Hip angle, Ankle angle and Hamstring force. The critical thresholds for each hypothesis test are shown as $F^{*}$ on each graph. Exceeding this number is evidence of the trueness of null hypothesis which is the existence of learning effect in this test.

**Supplementary Figure 3.** Illustration of a dual belt instrumented treadmill including (a) footprint of right and left leg on each belt during a step, and scenarios where part of one foot (b) or both feet (c) contact one single belt. Figure (b) and (c) show the scenarios where part of one leg or both legs land on one single belt. Scenario (c) is referred to as “cross stepping” throughout this paper. Data from the latter two scenarios were excluded.

**Supplementary Figure 4.** Hybrid gait event detection algorithm
