# Supplementary figures and images for: The role of the ankle plantar flexor muscles in trip recovery during walking: a computational modeling study
Source: Front Sports Act Living. 2023 Jul 18;5:1153229. doi: 10.3389/fspor.2023.1153229 (PMC10390771; doi:10.3389/fspor.2023.1153229)

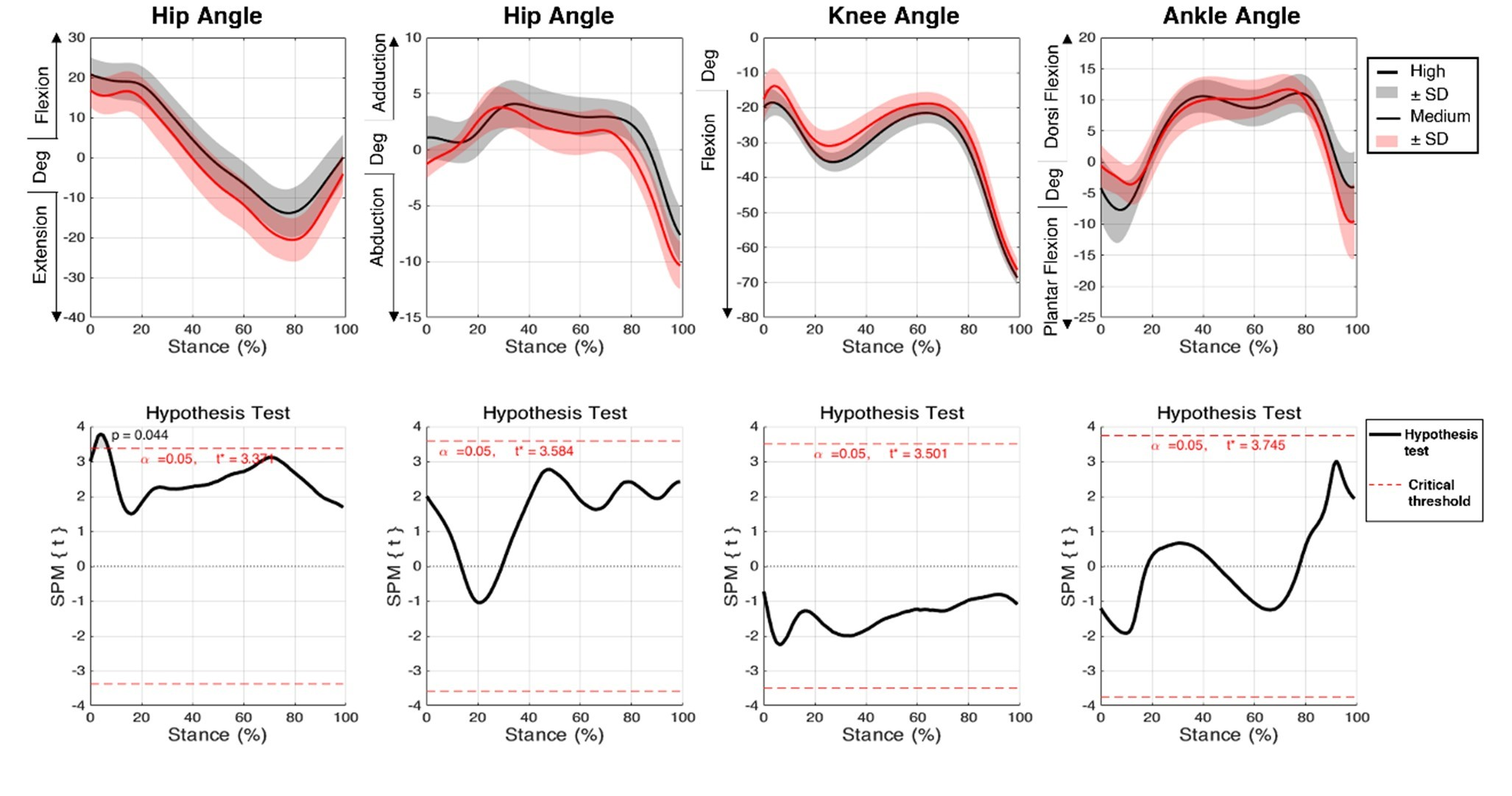

Supplement: Supplementary file 1 [file Presentation1.zip › SupplementaryFigure1a.jpg]

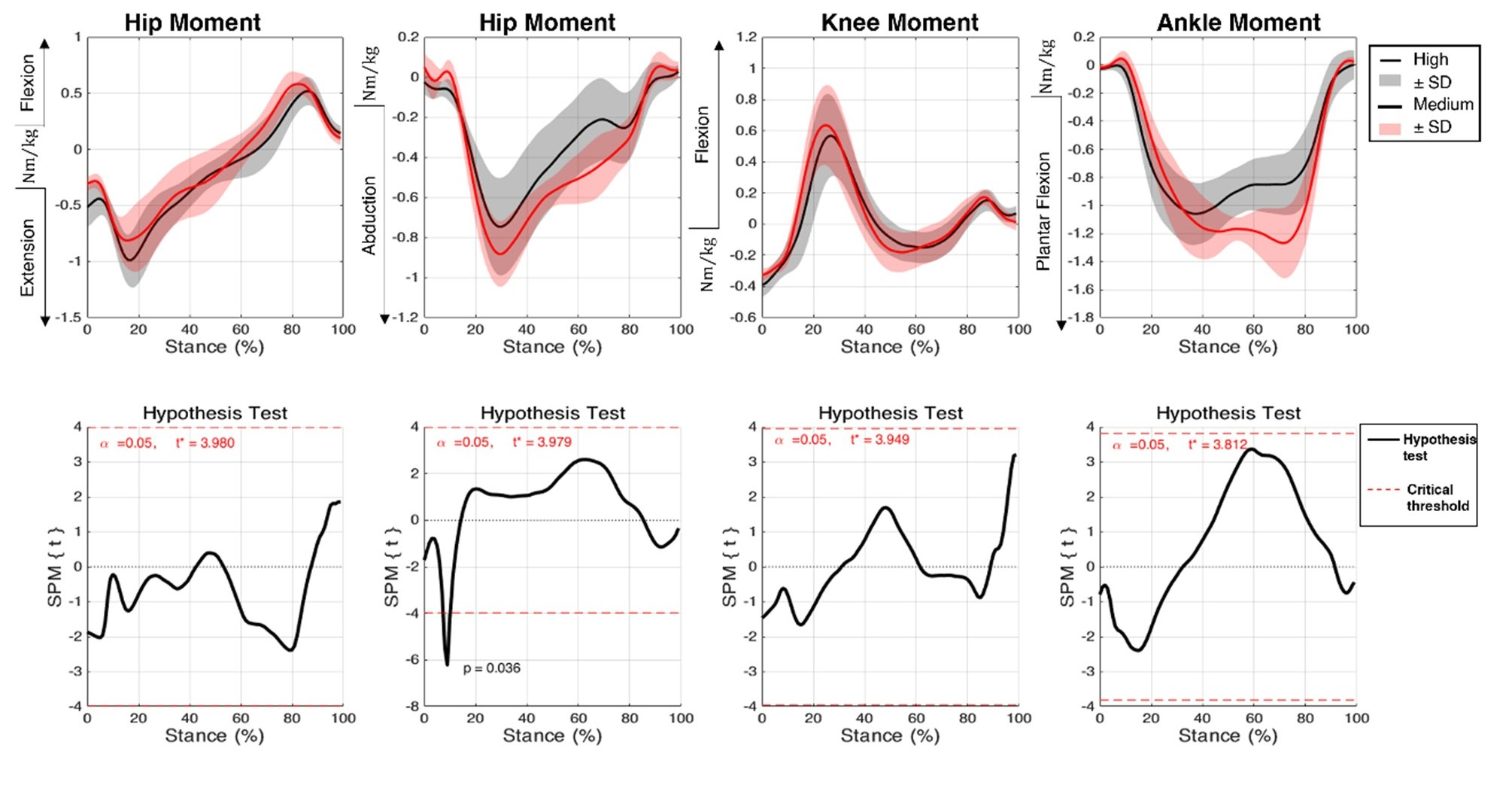

Supplement: Supplementary file 1 [file Presentation1.zip › SupplementaryFigure1b.jpg]

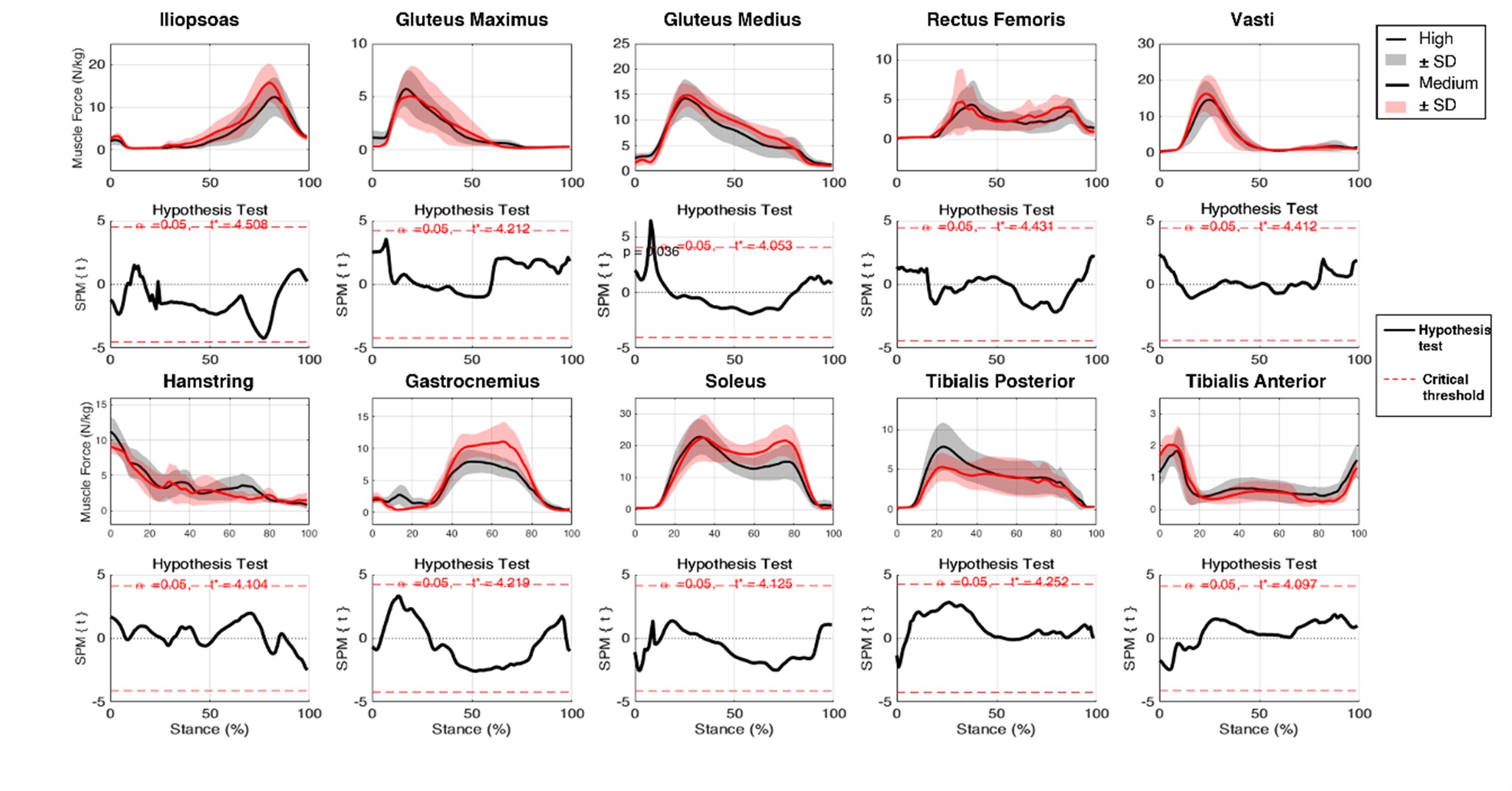

Supplement: Supplementary file 1 [file Presentation1.zip › SupplementaryFigure1c.jpeg]

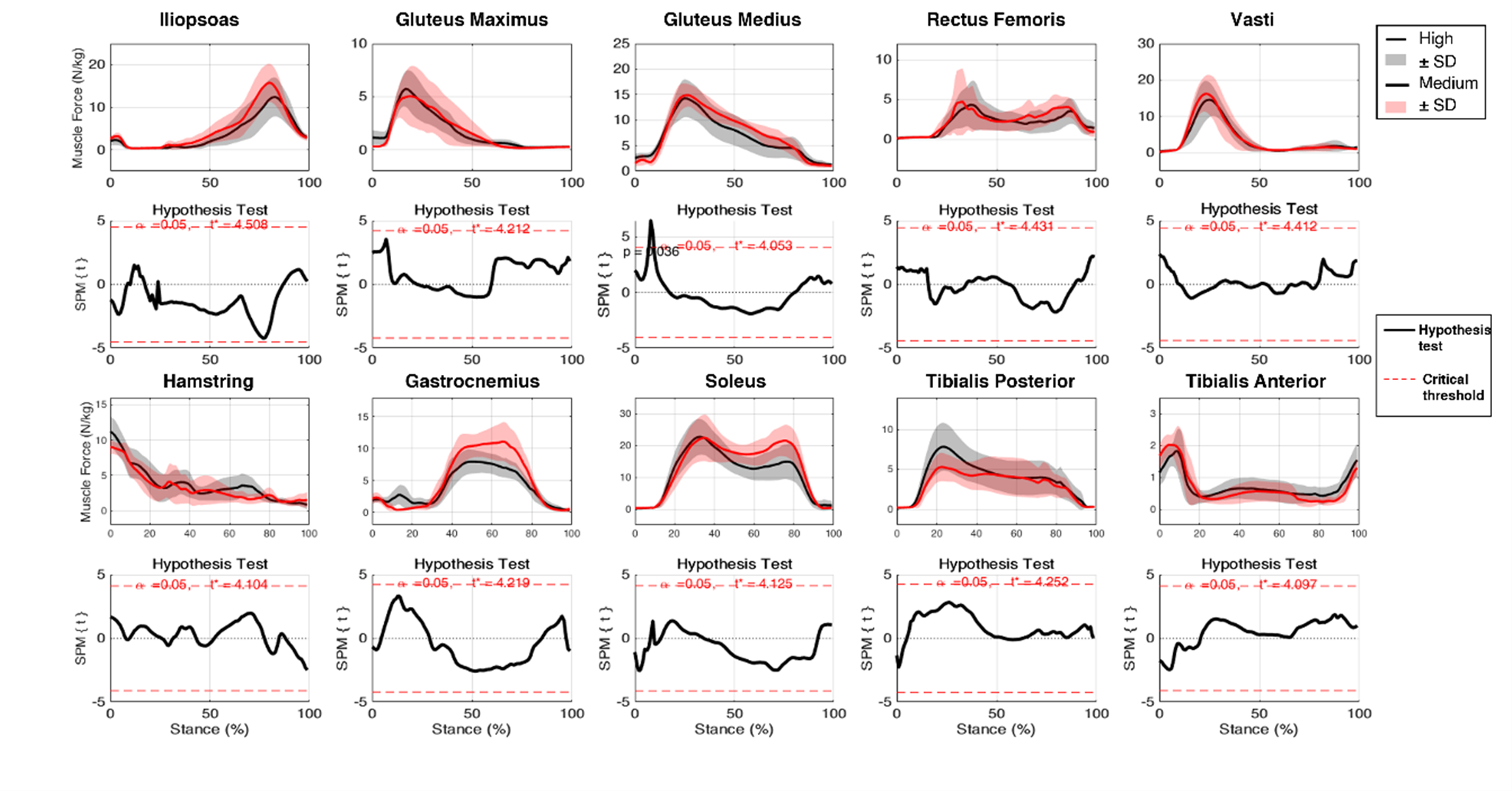

Supplement: Supplementary file 1 [file Presentation1.zip › SupplementaryFigure1c.png]

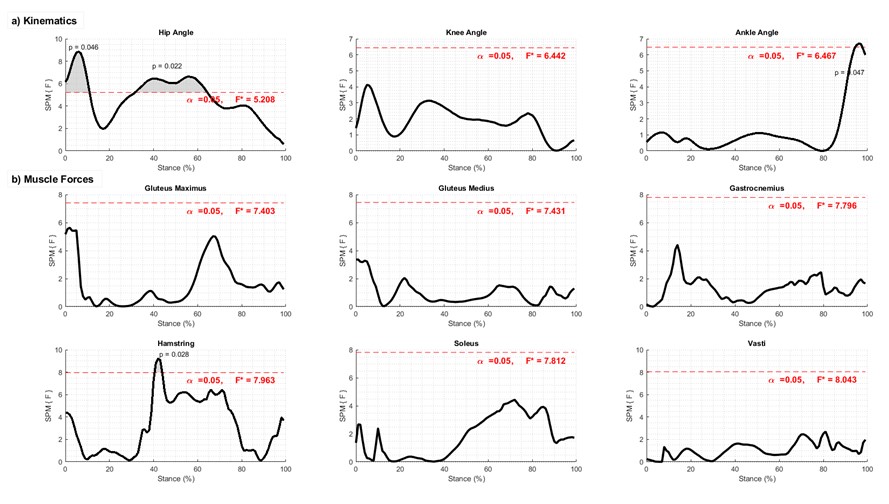

Supplement: Supplementary file 1 [file Presentation1.zip › SupplementaryFigure2a.jpg]

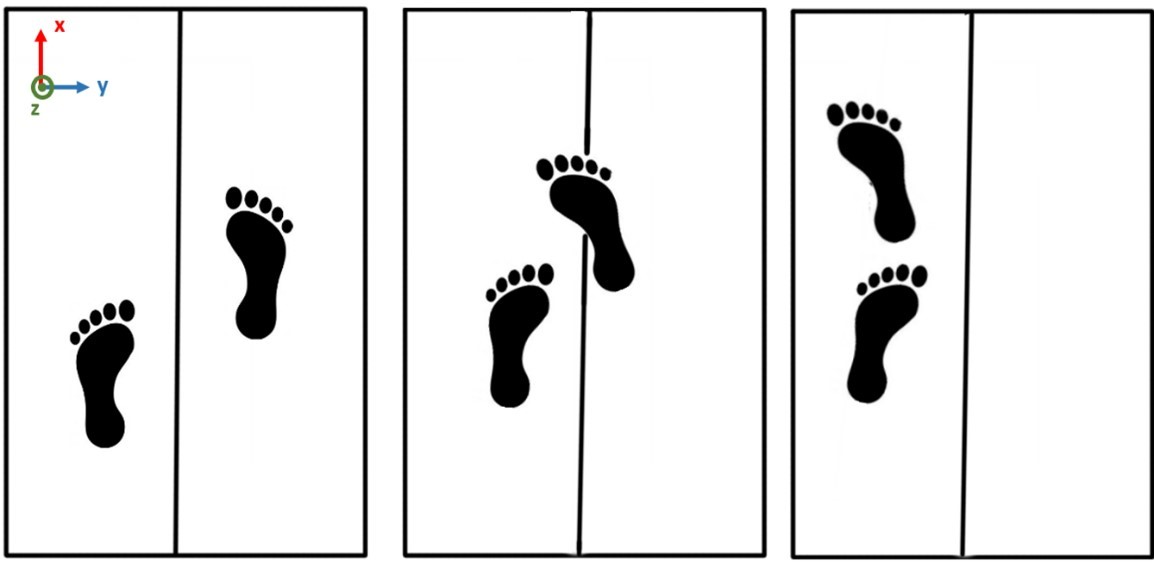

Supplement: Supplementary file 1 [file Presentation1.zip › SupplementaryFigure3.jpg]

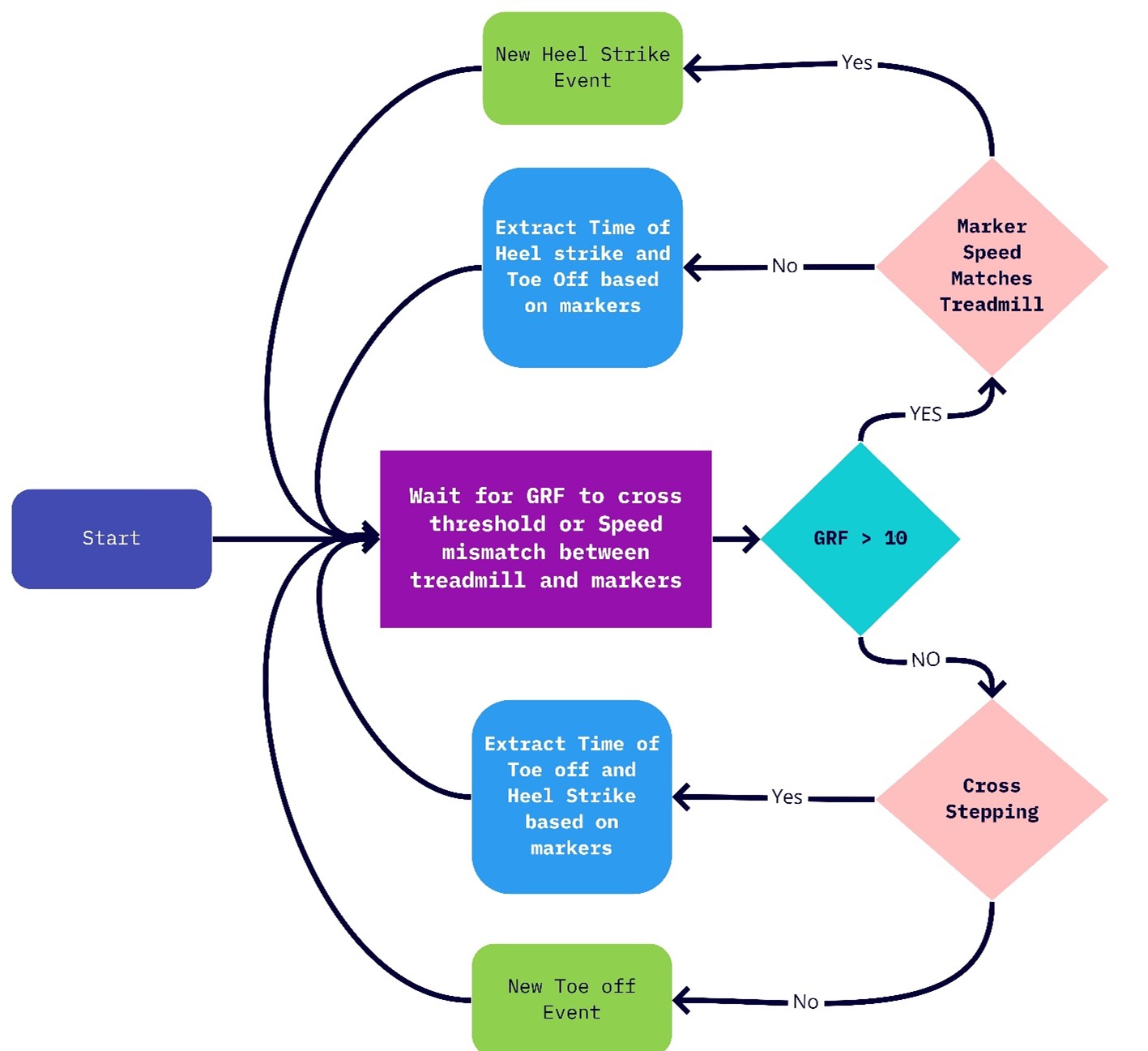

Supplement: Supplementary file 1 [file Presentation1.zip › SupplementaryFigure4.jpg]
